# Timbe (*Acaciella angustissima*) Pods Extracts Reduce the Levels of Glucose, Insulin and Improved Physiological Parameters, Hypolipidemic Effect, Oxidative Stress and Renal Damage in Streptozotocin-Induced Diabetic Rats

**DOI:** 10.3390/molecules23112812

**Published:** 2018-10-30

**Authors:** Adriana Jheny Rodríguez-Méndez, Wendy Carmen-Sandoval, Consuelo Lomas-Soria, Ramón G. Guevara-González, Rosalía Reynoso-Camacho, María Elena Villagran-Herrera, Luis Salazar-Olivo, Irineo Torres-Pacheco, Ana A. Feregrino-Pérez

**Affiliations:** 1 Facultad de Medicina, Universidad Autónoma de Querétaro, Clavel No 200, Col. Prados de la Capilla, 76176 Querétaro, Mexico; adjen.rm@gmail.com (A.J.R.-M.); wendycarmensandoval@hotmail.com (W.C.-S.); marielcosa@hotmail.com (M.E.V.-H.); 2 División de Estudios de Posgrado, C.A. Ingeniería de Biosistemas. Facultad de Ingeniería. Universidad Autónoma de Querétaro, C. U. Cerro de las Campanas, S/N, 76010 Querétaro, Mexico; cons_soria@hotmail.com (C.L.-S.); ramonggg66@gmail.com (R.G.G.-G.); Irineo.torres@uaq.mx (I.T.-P.); 3 Departamento de Investigación y Posgrado en Alimentos, PROPAC, Facultad de Química, Universidad Autónoma de Querétaro, C. U. Cerro de las Campanas, S/N, 76010 Querétaro, Mexico; rrcamachomx@yahoo.com.mx; 4 Instituto Potosino de Investigación Científica y Tecnológica, IPICYT, camino a la presa san José 2055, col. Lomas 4 sección, 78216, San Luis Potosí, Mexico; olivo@ipicyt.edu.mx

**Keywords:** *Acacciella angustissima*, streptozotocin-diabetic rats, antidiabetic effect, polyphenolic, protocatechuic acid

## Abstract

In Mexico one in 14 deaths are caused by diabetes mellitus (DM) or by the macro and microvascular disorders derived from it. A continuous hyperglycemic state is characteristic of DM, resulting from a sustained state of insulin resistance and/or a dysfunction of β-pancreatic cells. *Acaciella angustissima* is a little studied species showing a significant antioxidant activity that can be used as treatment of this disease or preventive against the complications. The objective of this study was to explore the effect of oral administration of *A. angustissima* methanol extract on physiological parameters of streptozotocin-induced diabetic rats. The results indicated a significant reduction in blood glucose levels, an increase in serum insulin concentration, a decrease in lipid levels and an improvement in the parameters of kidney damage by applying a concentration of 100 mg/Kg B.W. However, glucose uptake activity was not observed in the adipocyte assay. Moreover, the extract of *A. angustissima* displayed potential for the complementary treatment of diabetes and its complications likely due to the presence of bioactive compounds such as protocatechuic acid. This study demonstrated that methanol extract of *Acacciella angustissima* has an antidiabetic effect by reducing the levels of glucose, insulin and improved physiological parameters, hypolipidemic effect, oxidative stress and renal damage in diabetic rats.

## 1. Introduction

Hyperglycemia is a feature of diabetes mellitus (DM), resulting in a sustained state of insulin resistance and/or a dysfunction secreting of β-cells of the pancreas [1]. Diabetes is one of the major chronic diseases worldwide, affecting over 150 million people worldwide, and it is estimated that by 2025 this amount will double [2].

According to the World Health Organization Mexico ranks ninth in DM prevalence worldwide, representing the first cause of death in the country [3]. Additional consequences of diabetes include macro and microvascular complications derived from the characteristic oxidative stress of the disease. This oxidative stress occurs because of glucose being oxidized which leads to the formation of reactive oxygen species (ROS). An in vivo model of oxidative stress occurs after injecting streptozotocin (STZ), causing toxic effects through damaging β-cells of the pancreas [2]. Worldwide, plant extracts have been used as complementary or alternative treatments in patients with DM, an activity that has been documented since ancient times in developing countries [4]. Its efficiency has been attributed to the presence of polyphenolic compounds present in various plants and foods, whose antioxidant activity contributes with scavenging free radicals and therefore prevents DM [5]. Moreover, the hypoglycemic activity of these plants is also due to the presence of compounds such as alkaloids, terpenes, flavonoids, saponins, among others, that have been proposed to possess insulinomimetic activity, however their mechanism of action is unknown [6].

In Mexico, *Acaciella angustissima*, known as timbe, timbre, cantemo and guajillo [7], is often used as fodder for livestock, as a natural fuel and in the fur industry as a vegetable-based tanner. *A. angustissima* plants reach 2–5 m in height and the brown pigmented 3.5 to 8 cm long pods reach maturity between the months of November-February [8]. The presence of bioactive compounds in *A. angustissima* has been documented, suggesting this plant as a natural source of antioxidants capable of scavenge the formation of ROS derived from the hyperglycemic state characteristic of DM, in addition some species that have antioxidant capacity also provide protection against destruction or low activity of β-cells [9]. Some biological properties of *A. angustissima* pods have been reported [8], however, little is known about medicinal properties of this species. It is expected that the presence of bioactive compounds in the plant and the results derived from the present work could generate information to use *A. angustissima* pods as a natural coadjuvant alternative in diseases related to oxidative stresses such as DM. The objective of this study was to explore the effect of oral administration of *A. angustissima* methanol extract on the physiological parameters of streptozotocin-induced diabetic rats. 

## 2. Results and Discussion

### 2.1. Phenolic Compounds and Antioxidant Capacity of Methanolic Extract (MEA)

Plants biosynthesize a myriad of biologically active compounds, which, when used in adequate non-toxic doses, are capable of regulating glucose levels, as well as alleviating oxidative stress [1,3,4,5]. Reduction of hyperglycemia is one of the main objectives in diabetes, as well as the reduction of derived complications, i.e., renal dysfunction or nephropathy. Several plants that have been used to reduce hypoglycemic effects and used as alternative medicine therapies are for instance *Coriandrum sativum* and *Cnidoscolus chayamansa* [4,5]. The hypoglycemic effect in various plants has been attributed to the presence of compounds such as polyphenols given their antioxidant properties [10,11,12]. 

Table 1 shows the quantification of phenolic compounds and antioxidant capacity. The methanol extract (MEA) obtained by maceration [13] reached 3.5 times higher concentration than the aqueous extract (W) obtained by Soxhlet [5], both in a ratio 1:10 (*w*/*v*), with values of 121.75 ± 2.46 and 36.68 ± 0.70 mg eq. Gallic acid/g timbe extract, respectively, for total phenols. The content of total flavonoids and condensed tannins showed significant differences between MEA and W of timbe pods (Table 1). The results indicated that methanolic extracts of *A. angustissima* pods are a good source of polyphenolic compounds as shown in Table 1. These results agree with those reported by Vargas-Hernández et al. [14]. 

The presence of high concentrations of phenolic compounds such as total phenols and flavonoids suggest a close relationship with the presence of antioxidant capacity according to various studies [3,11,12]. In addition, it has been documented that polyphenols counteract the oxidative stress that can lead to pathologies such as atherosclerosis, cancer and diabetes [5,6]. Polyphenols are multifunctional as they are reducing agents, hydrogen-donating antioxidants, and singlet oxygen quenchers. Furthermore, strong evidence has been provided for an indirect antioxidant activity of polyphenols exerted by activating the endogenous defense system. In addition, several experimental data indicate a tight relationship between glutathione (GSH) and its related enzymes and dietary polyphenols. This suggests that the presence of polyphenols induces GSH to act as an enzymatic substrate for transferases involved in the detoxification/reduction of H_2_O_2_, lipid hydroperoxides and electrophilic compounds [15]. The results agree with this tendency when showing a high antioxidant capacity by the 2,2-azino-bis (3-ethylbenzothiazoline-6-sulphonic acid) (ABTS) radical cation assay method for MEA than the aqueous extract (Table 1). Antioxidant activity of W (15.88 ± 1.94 mg eq. Trolox/g of sample) of *A. angustissima* was lower when compared to MEA (70.34 ± 13.96 mg eq. Trolox/g of sample; *p* ≤ 0.05).

Although this species has not been widely studied, it is known that another species of the genus Acacia, known as *A. pennatula* shows high antioxidant activity, which is directly related to the content of phenolic compounds that confer it a high antioxidant potential [13]. In this context, the antioxidant capacity in vitro using ABTS, with a preliminary test to evaluate compounds having electron-donating and/or proton-free radical quenching properties and the inhibition of oxidative processes based on the above results (Table 1), it was possible to demonstrate that MEA may be a potential source of antioxidant compounds and these results suggest that this activity could inactivate the radicals generated in a body in the state of hyperglycemia. Studies suggest that antioxidant capacity contributes positively in the prevention and treatment of diabetes mellitus (DM) [16], making essential to know the content of phenolic compounds present in plants extracts to evaluate their hypoglycemic activity.

### 2.2. HPLC Analysis

**Figure 1:**
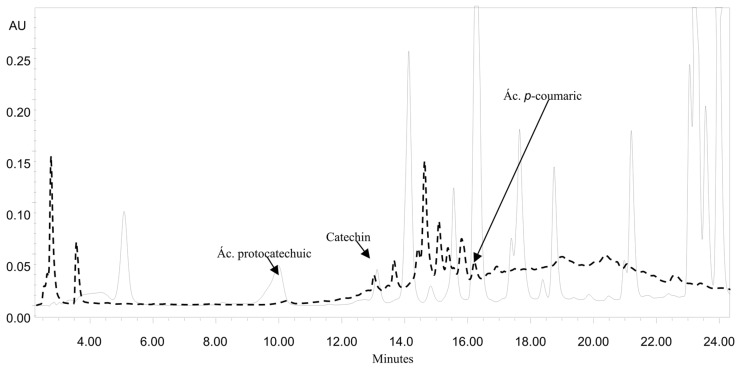
HPLC chromatogram of MEA: in black HPLC separation of MEA phenolics; and in dotted HPLC profile of phenolics standards (data were recorded at 280 nm).

Phenolic compounds are a broad range of compounds synthesized by plants as part of their secondary metabolism. They can be categorized into different classes such as stilbenes, flavonoids, lignans and phenolic acids [17]. The MEA extracts of *A. angustissima* showed the presence of protocatechuic acid, catechin and coumaric acid in quantitative HPLC analysis (Figure 1). Catechin and coumaric acid were not quantified due the low retention time of the compounds on the column. Protocatechuic acid showed values of 197.33 (MEA) and 178.33 (W) µg/g of sample. The concentration of protocatechuic acid was the highest based on the comparison of the retention times and areas of the standards used. Protocatechuic acid is a major metabolite of complex polyphenols, especially anthocyanins [4]. Protocatechuic acid has been attributed anti-inflammatory properties by decreasing release of interleukin (IL)-1 beta, tumor necrosis factor–alpha (TNF-) and prostaglandin E2 in brain, as well as antioxidant and free radical scavenging activities, peroxidation inhibition and estrogenic/antiestrogenic and antihyperglycemic activity by decreasing ROS levels, protein carbonyl, carboxymethyllysine pentosidine, sorbitol, fructose and methylglyoxal [18,19]. Moreover, in a diabetes model (streptozotocin-induced diabetic rats), protocatechuic acid prevented coagulation and inflammation by inhibiting the plasma levels of the plasminogen activator inhibitor 1 (PAI-1), antithrombin III (AT-III), protein C, C-reactive protein (CRP), and von Willebrand factor (vWF) and reduced IL-6, TNF-α, and monocyte chemoattractant protein-1 (MCP-1) levels in the heart and kidneys [20]. The inhibition of lipid mediator generating enzymes is one of protocatechuic acid anti-inflammatory mechanisms. Protocatechuic acid inhibits COX-2 and PGE_2_ expression both in vitro in LPS-treated RAW264.7 cells and *in vivo* in mice treated with carrageenan, a polysaccharide that induces inflammation [15]. These results suggest that the anti-inflammatory effects of protocatechuic acid might be beneficial in various chronic degenerative diseases such as diabetes in which the inflammatory process plays an important part in the pathogenesis. In another species of Acacia, *A. confusa* a wide range of phenolic compounds, especially flavonoids, flavonol glycoside and phenolic acid derivatives were the main phytochemicals isolated from different plant organs [21]. Despite the fact the amount of catechin detected in our study was low, it has been documented that similar low catechin concentrations contributed to the reduction of obesity-related diabetes [22]. While in the study reported by Amalan [23] it was observed that the administration of *p*-coumaric acid at a dose of 100 mg/kg b.w. in diabetic rats (induction with streptozotocin, 40 mg/kg b.w.) decreased glucose and lipid levels through GLUT2 activation of the pancreas. These data suggest that the MEA contains a mixture of compounds that influence the metabolism of glucose and lipids, generating hypoglycemic activity.

### 2.3. Effect of A. angustissima in Serum Glucose and Insulin Levels

In the streptozotocin-induced diabetes model it has been reported that this drug causes a deficit of antioxidants and increased oxidative stress, causing damage and partial destruction of pancreatic β-cells, generating a decrease in insulin production similar to what occurs in type 1 diabetes, causing a reduction in glucose uptake in peripheral tissues such as muscle and adipose tissue, as well as an increase in blood glucose, which leads to the development of a diabetic condition [2,24,25]. Induction of experimental diabetes via the streptozotocin led to significant (*p* < 0.05) increase in blood glucose as showed in Table 2. According to glucose levels and insulin in the groups of study, untreated diabetic animals showed significantly elevated amounts of glucose (372.6 ± 6.05 mg/dL) and a reduction in serum insulin concentrations (7.0 ± 0.01 µU/mL) when compared to healthy control and MEA control (Table 2). However, the administration of MEA at the highest concentration (100 mg/kg) significantly restored the glucose (146.2 ± 4.03 mg/dL) and insulin (18.1 ± 0.02 µU/mL) levels in diabetic rats near to normal levels (86.8 ± 2.05 mg/dL and 28.2 ± 0.1 µU/mL, respectively). The administration of MEA at a concentration of 100 mg/kg showed better results than those obtained by administration with glibenclamide, a hypoglycemic drug of the sulfonylureas class that stimulates insulin secretion by pancreatic β cells according to the data reported by Almuaigel et al. [26], where glibenclamide (600 µg/kg body weight) was administered to diabetic rats, generating a reduction in glucose levels (312.30 ± 20.59 mg/dL) with respect to diabetic control (487.9 ± 40.67 mg/dL), however presenting values of almost two times more than healthy control (119.50 ± 12.90 mg/dL). Notwithstanding, the extract of MEA at the highest concentration, displayed better results in relation to the reduction of glucose levels in diabetic rats (Table 2). The latter results suggest that at least partially hypoglycemic effect shown by MEA could be attributed to the phenolic concentration reported in the extract of *A. angustissima*. Diabetic rat studies have demonstrated the efficacy of the phenolic compound, exhibiting a lowering effect of glucose and antioxidant activity ten times greater than vitamin E, demonstrating that this compound has a very important biological activity [27]. For example, the supplementation of protocatechuic acid at 2% and 4% in diabetic mice for 12 weeks was useful in preventing glycation associated with diabetes. The presence of protocatechuic acid in mice decreased water intake, food intake, urine volume, plasma BUN level, HbA_1C_ level, urine glycated albumin, urinary albumin, renal activity and mRNA expression of PKC-α and PKC-β and RAGE mRNA expression. Moreover, increased body weight, plasma insulin levels, creatinine clearance rate, renal activity and expression of GLI, suggesting that protocatechuic acid had an antihyperglycemic, antiglycative and renoprotective effects via increasing plasma insulin, reducing plasma glucose, reducing renal level of glycation end products, fibronectin, TGF-β, and repressing renal activity and expression of AR, SDH, GLI, PKC-α, PPAR-γ, restoring PPAR-γ, and suppressing RAGE [28]. In other studies, it is proposed that protocatechuic acid in combination with cyaniding-3-*O*-β-glucoside can activate PPAPγ exerting a similar activity to that in which regulates the activation of GLUT4 [29]. Moreover, oral administration of protocatechuic acid (50 and 100 mg/kg/day) in diabetic rats with STZ significantly decreased blood pressure and vascular reactivity, as well as reactivation of enzymatic antioxidant activity, proposing protocatechuic acid as a protective agent against the complications of diabetes such as nephropathy [20]. Harini and Pugalendi [27] showed that administration of 50, 100 and 200 mg/kg/day of protocatechuic acid for 45 days induced decreased plasma glucose levels, HbA_1C_ levels, activity of glucose 6-phosphatase and fructose 1,6-diphosphatase in liver, reduced adipose tissue of diabetes mellitus pancreas and normalized pancreatic islets, increased plasma insulin levels, hexokinase activity and increased glycogen content in liver, suggesting that the mechanism of protocatechuic acid is exerted antihyperglycemic effects by restoring carbohydrate metabolic enzyme activity and increasing plasma insulin levels. Thus, protocatechuic acid might exert a potential antihyperglycemic effect that is comparable with that obtained with classical antidiabetic drugs such as gliblenclamide. In other experiment, Yüksel et al. [30] have demonstrated that protocatechuic acid has a protective effect on renal damage by reducing oxidative stress and tissue damage and Safaeian et al. [31], demonstrated that protocatechuic acid prevented hypertension (another coexisting complication of diabetes), reduced plasma H_2_O_2_ concentration and increased superoxide dismutase activation. Taking together these data suggests a possible molecular mechanism for protocatechuic acid action. The protocatechuic acid is known that diminishes the mRNA expression and activity of PKC-α and PKC-β, and, consequently, of TGFβ1 that, in turn, promotes ECM formation and tissue fibrosis. Additionally reverts the PPARγ and, particularly, PPARγ, that is a main metabolic regulator of glucose and lipid metabolism, the most extensively studied and clinically validated gene for therapeutic utility in diabetes, and the main target for many antidiabetic drugs such as thiazolidinediones. The studies have demonstrated that protocatechuic acid is able to increase glucose uptake and enhancing GLUT4 translocation as well as adiponectin secretion in human primary omental adipocytes [29]. Insulin activity, possibly resulting from the increased activity of PPARγ induced by protocatechuic acid. Currently, there is a search for PPARγ ligands that do not present unwanted side effects, in this sense *A. angustissima* extract could offer an interesting possibility in diabetes care. This scientific evidence, together with the data shown in this paper, suggests that the hypoglycemic effect shown by MEA may be due to the presence of phenolic compounds such as protocatechuic acid. 

The results showed significantly elevated amounts of glucose and a reduction in serum insulin concentrations in untreated diabetic animals when compared to healthy control and MEA control. Reversible effect with the administration of MEA at the highest concentration (100 mg/kg) significantly restored the glucose and insulin levels in diabetic rats near to normal levels. Other studies in plants indicate that administrations of 100 and 200 mg/kg of the seed, peed powder and aqueous extract of the leaves of *Abelmoschus sculentus* decreased blood glucose in STZ-induced diabetic rats [32,33]. A possible mechanism by which these compounds from MEA exert their hypoglycemic action in diabetic rats could involve the increase of pancreatic secretion of insulin from the existing β-cells [12]. In our study, there was an increment in insulin levels up to 64% in the group of diabetic + 100 mg/kg MEA. Most of these improvements may be due to the content of bioactive compounds such as phenolic compounds, found in adequate proportion for this species in previous studies showing a hypoglycemic effect [33].

### 2.4. Physiological and Biochemical Evaluations

The decrement in body weight observed in uncontrolled diabetic rats may be the result of protein loss due to the inability to use carbohydrates as an energy source, or to excessive diuresis featured in diabetic tables. The results indicated a decrease in the body weight of diabetes control (272 ± 15 g) compared to healthy control (304 ± 20 g; *p* < 0.05). The group of diabetic rats with MEA (100 mg/kg) increased their weight (281 ± 8 g), without presenting significant difference with respect to diabetic control (Table 3), these values likely due to a high antioxidant activity of phenolic compounds present in the extract supporting the reduction of glycemic. It has been documented that an increase in the weight of the kidney in diabetic animals compared to healthy ones can be attributed to an increase in early cell proliferation in a compensatory way [27]. The results indicated a significant decrease in the weight of the kidneys of diabetic rats once administered orally MEA, reaching values comparable with healthy control. This result suggested a protective effect of MEA on glomerular cells in diabetes induced by STZ in rats. 

When a histological analysis of the kidney was performed (data not shown), no difference was found between the controls and the treatment groups, indicating that it is necessary to increase the exposure time to observe histological damage at the renal level, but not for the biochemical parameters. Renal dysfunction displayed elevated levels of urea, creatinine and protein, that are considered as markers of this condition. The results in Table 3, on one hand showed a significant increase (*p* <0.05) in the urine levels of urea (50.4 ± 22.7 mg/dL), creatinine (0.70 ± 0.18 mL/min) and protein (1.05 ± 0.01 mg/24 h) of diabetic rats compared to healthy control rats (7.2 ± 4.0 mg/dL, 0.57 ± 0.01 mL/min, 0.52 ± 0.03 mg/24 h, respectively). On the other hand, treatment with MEA (100 mg/Kg) displayed levels of urea (18.1 ± 6.5 mg/dL), creatinine (0.46 ± 0.09 mL/min) and protein (0.54 ± 0.05 mg/24 h) that were similar to healthy control. 

Studies with several species such as C. esculenta [34], B. aegyptiaca [35], Panax ginseng [36] and the herbal formulation D-400 [37] showed similar behaviors in rats induced with STZ. In the same way, the administration of garlic extract will also decreased serum urea and creatinine level due to reduction of activity of xanthine oxidase and lipid peroxidation which participates in oxidative degradation of lipid [38]. Therefore, the content of bioactive compounds in MEA represents an important source of antioxidants, in addition to decrease complications of diabetes such as renal dysfunction and hyperlipidemia [32].

### 2.5. Determination of Serum Lipid Profile

Rats induced by STZ display hyperglycemia, hypercholesterolemia and hypertriglyceridemia. Hyperlipidemia is a characteristic marker of the diabetic state and might be considered a result of the hormone uninhibited lipolytic fat deposits [39,40]. The effect of MEA on serum lipids of the tested groups is shown in Table 4. The levels of TG, TC and LDL in the group of diabetic + MEA (100 mg/kg) were 64.5 ± 0.025, 72.9 ± 2.8 and 17.6 ± 3.9 mg/dL, respectively, which were significantly lower (*p* < 0.05, 0.01) than diabetic control rats (72.4 ± 4.8, 104.2 ± 2.1 and 51.9 ± 4.3 mg/dL, respectively). The diabetic + MEA-treated group (100 mg/kg) had also a significant increased response to elevated levels of HDL-C next to healthy control rats, compared with diabetic control rats.

Studies indicated that a resistance to insulin characteristic of diabetes, increased the concentration of serum glucose and lipids, generating oxidation states of these particles that resulted in an increase in the synthesis of cholesterol and triglycerides, as well as a decrease in the use of glucose by erythrocytes, inducing patients and animal models to a state of hyperlipidemia (another complication of DM) [41]. 

The use of plants to counteract not only the sustained increase in blood glucose, but also the problems of hyperlipidemia is common in several countries. The results of the present study were consistent with the work of Akhtar et al. [42] showing that *Catharanthus roseus and Coccinia cordifolia* displayed a lipid-lowering effect. Moreover, ethanolic extract of *Aloe vera* leaf, with doses of 300 mg/kg showed increased levels of insulin from regenerated pancreatic beta-cells. Besides, the plasma lipids, liver and kidney triglycerides (TG) levels of the tested diabetic rats were also reduced after the administration of *Aloe vera* extract [43]. 

Reduction of blood colesterol level also shown in garlic extract may be due to hydroxymethyl glutaryl-CoA reductase inhibition which suppresses the colesterol producing metabolic pathway [38]. The antihyperlipidemic effect found in this study, might be attributed to the presence of the bioactive compounds of the extract, such as protocatechuic acid. Borate et al. [44] showed that the administration of protocatechuic acid to hyperlipemedic rats at doses of 25 and 50 mg/kg significantly decreases serum levels of TC, TG and LDL and HDL significantly increased. This study proposed that the possible mechanism of action of protocatechuic acid is by the activation of the hepatic LDL receptor or exert an effect on the enzymes involved in the metabolism and excretion of cholesterol.

### 2.6. Determination of Lipid Peroxidation and Protein Content in Kidney

**Figure 2:**
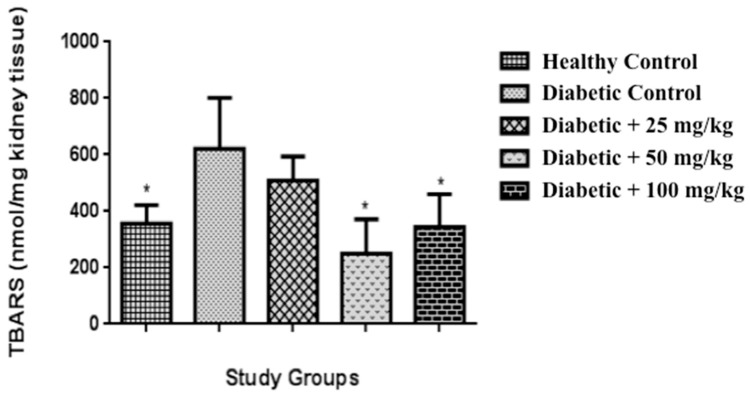
Effect of MEA on lipid peroxidation in kidney of diabetic rats. Data are expressed as mean ± SD for eight determinations. * *p* < 0.05 compared to diabetic control.

The substances that react with thiobarbituric acid (“TBARs”) assay has always been considered as a good “analytical marker” for oxidative stress, which results in an intensive lipid peroxidation of biological membranes. The high concentrations of lipid peroxides can propagate oxidative damage by increasing peroxide and hydroxyl radicals, contributing to functional impairment of various organs [45,46]. The present study has revealed that diabetic control rats showed an increase of TBARS compared to healthy control group (*p* < 0.05). The groups of diabetic of 50 and 100 mg/kg, respectively were able to reduce significantly the levels of TBARS (Figure 2), compared to diabetic control (*p* < 0.05). MEA administration decreased TBARS levels, suggesting that the extract possesses antioxidant principles that produce such responses, suggesting that MEA has protective effect on the oxidative stress in diabetic rats. A similar observation was reported in the administration of a *Punica granate* extract [47] in which, according to the author, the extract may be effective in correction of hyperglycemia and in the prevention of diabetic complications, Sroka and Cisowski [48] indicated that protocatechuic acid (0.05 and 0.10 mg/mL) increased the inhibition of lipid peroxidation and scavenging of H_2_O_2_ as well as scavenging of DPPH.

### 2.7. Glucose Incorporation Assay in Adipocyte Cells

**Figure 3:**
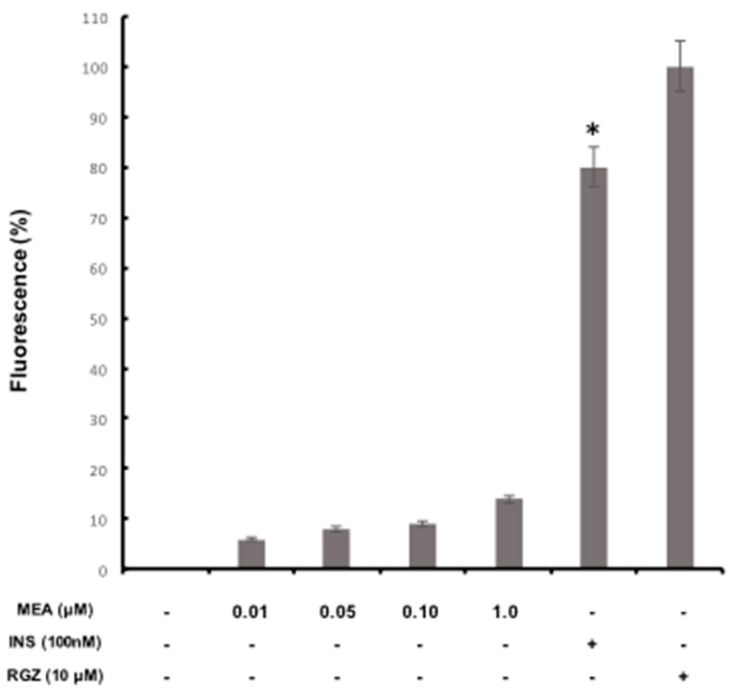
Effect of MEA on glucose uptake activity in mature adipocytes 3T3-F442A. Control treatments were incubated with insulin 100 nM (INS) or rosiglitazone (10 μM (RGZ). The results are presented as the mean ± S.D. of three independent experiments in triplicate. * *p* < 0.05 compared to rosiglitazone control

Previous studies have reported that insulin helps the increase of glucose uptake in adipocyte cells [49]. This research used the same concentration of insulin according to reports showing that concentration of 100 nM insulin increased glucose uptake significantly by about 1.64 times in 3T3F442A adipocyte cells, which were also used to stimulate glucose uptake in the presence of MEA [49,50]. The effect of MEA on glucose uptake activity in adipocytes 3T3F442A was as follows (Figure 3). Insulin (100 nM) alone showed a significant difference (*p* < 0.05) increased glucose uptake by 80% compared to control of rosiglitazone maleate (RGZ), whereas for negative control was an increase of 100%. Furthermore, MEA (0.1, 0.5, 0.10 and 1.0) had no effect on glucose uptake activity in adipocytes 3T3F442A, compared to positive control (insulin). The null glucose uptake cause by MEA indicated that the extract either did not increase the uptake of glucose by the action of insulin sensitization, or acted as insulin mimetics, although there are studies showing that the uptake of insulin likely is caused by one or both above mentioned mechanisms as is the case of *Lagerstroemia speciosa* [49] and *Agaricus campestris* [51]. Improved glucose uptake has been reported in an insulin-mimetic, *Salvia miltiorrhiza* through mechanisms related to insulin sensitization, while in *Campsis grandiflora* [52] and *Vaccinium angustifolium* [53] through both mechanisms. One of the possible antidiabetic mechanisms that MEA could perform, would be either the increase of pancreatic insulin secretion from β-pancreatic cells existing [11] or that this extract acts as regenerator stimulating β-cell insulin secretion [2].

## 3. Material and Methods

### 3.1. Collection of Material and Preparation of Extract

The pod collection was carried out in La Cañada, Querétaro, Mexico (20°36′34′′ N, 100°20′20′′ O and 1875 m a.s.l.) in December 2010. A specimen of the plant was identified and deposited in the Ethnobotanical collection of the Herbarium of QMEX, School of Natural Sciences, Universidad Autonoma de Queretaro. The pods were air-dried and the seeds were separated from the pod. The pods were ground using 40-mesh powder (Scientific Apparatus, Philadelphia, PA, USA). The material was extracted using methanol as extraction solvent in a ration (1:10 *w*/*v*) by maceration (M) [13] and by Soxhlet (S) [5]. Methanolic extract from *A. angustissima* pods (MEA) was evaporated to dryness in vacuum for completed removal of solvent and the extracts were stored at 4 °C for further use.

### 3.2. Phenolic Compounds and Antioxidant Capacity of Methanolic Extract (MEA)

Total phenolic content was determined by the Folin-Ciocalteu method [54]. Tannins were analyzed with the vanillin-HCl method according to the procedure of Deshpande [55]. Flavonoids were quantified by a method described previously [56]. The MEA antioxidant activity to scavenge free radicals was determined by the method of Van Den Berg et al. [57].

### 3.3. HPLC Analysis

Plant extracts were filtered through a 0.2 mm membrane and 20 μL were injected in triplicate into a reverse phase column (Zorbax Eclipse XDB-C18, 60 Å, 5 μm, 250 × 4.6 mm) using a Waters HPLC system (Waters Corporation, Milford, MA, USA). The mobile phase consisted of solvent A (acetonitrile) and solvent B (0.0125 N acetic acid). The elution was as follows: isocratic from 0 to 2 min with 5% A and 95% B, gradient condition from 2 to 5 min starting with 5% to 15% and ending with a gradient conditions 5 to 20 min starting with 15% A and ending with 50% gradient conditions of 20 to 25 min starting with 50% A and ending with 5% isocratic conditions of 25 to 35 min with 5% A and 95% B. The flow rate was 1 mL/min, the absorbance was measured at maximum length of 280 nm and 20 µL of sample were injected. Quantification was performed by external standardization using protocatechuic acid, gallic acid, caffeic acid, rosmarinic acid, *p*-coumaric acid, quercetin, naringenin, catechin, kaempferol, and rutin.

### 3.4. Experimental Protocol with Animals

The experimental protocol was developed in accordance with the ethical guidelines of the Animal University of Queretaro, based on the Mexican Official Law (NOM-062-ZOO-1999). At 6 weeks of age, 80 male Wistar rats (weighing 250–300 g) were obtained from Rismart S.A. de C. V., Mexico City. The animals were acclimated in individual cages for a week under the following conditions: 20–25 °C with a 12-h light-dark cycle with free access to water and a base diet (Zeigler NIH-31, Rismart S. A. de C. V.) containing 18% crude protein, 4% crude fat and 5% crude fiber and 3.5% mineral mix. Once the acclimatization was concluded, type 2 diabetic rats were induced by an intraperitoneal injection of freshly prepared solution of streptozotocin (STZ, Sigma Chemical Company, Saint Louis, MO, USA), in a single dose at a concentration of 45 mg/kg body weight; dissolved in a citrate buffer, 0.01 M, pH 4.5) after overnight food deprivation. Healthy rats received the same volume of vehicle (citrate buffer). Type 2 diabetic rats were determined at the 72 h after of the injection. The rats with fasting blood glucose ≥ 240 mg/dL were considered diabetic for this experiment.

### 3.5. Animal Grouping.

Once the induction of diabetes has been determined, the animals were divided into healthy and diabetics, and each of these groups were grouped randomly into groups of 10 rats each to give the groups shown in Table 5. Healthy and diabetic groups were fed with normal diet *ad libitum*. The healthy rats were assigned into four groups (10 rats per group) at random. Group 1 (healthy control (HC)) was given 0.1 mL/kg B.W./day of saline solution; Groups 2, 3, and 4 were administered whit MEA at a dose of 25, 50 and 100 mg/kg B.W./day respectively. Groups of STZ-induced diabetic rats were randomly divided into four groups (10 rats per group). Group 5 (diabetic control (DC)) was given 0.1 mL/kg B.W./day of saline solution; Groups 6, 7, and 8 were administered MEA at a dose of 25, 50 and 100 mg/kg B.W./day, respectively. The MEA was dissolved in water and administered orally. Blood glucose levels were determined weekly. The body weight was measured every week. One day before sacrifice animals were located in metabolic cages to obtain urine for determination of biochemical parameters related to DM. 

### 3.6. Collection of Blood and Tissue

After the experimental period (four weeks) the animals were sacrificed by anesthesia. Cardiac puncture was performed to obtain blood sample and then serum by centrifugation, dosed and stored at −80 °C until its use. The kidneys were dissected, washed with cold saline phosphate buffer, for subsequent freezing in nitrogen and storage at −80 °C until analysis.

### 3.7. Biochemical Evaluations.

According to Barham and Trinder [58], the serum glucose concentration was determined. Under fasting conditions and through commercial kit: serum insulin (Merck Millipore, Darmstadt, Germany), serum concentration of total cholesterol (TC), triglycerides (TG), low density lipoprotein (LDL) and high density lipoprotein (HDL)-cholesterol, (Genzyme Diagnostics, Pittsburgh, PA, USA), urine urea and creatinine clearance (Randox Laboratories Ltd., Northern Ireland, UK). Creatinine clearance (C_Cr_) was calculated on the basis of urinary Cr, serum Cr and urine volume using the following equation: CCr (mL/min) = [urinary Cr (mg/dL) × urine volume (mL)/serum Cr (mg/dL) × 1440 (min)].

### 3.8. Lipid Peroxidation and Protein Content in Kidney

Lipid peroxidation, was estimated by the method of Fraga et al. [59], and was expressed as thiobarbituric acid reactive substances (TBARS) µM/g of kidney tissue. The Bradford method was used for protein concentration as described in the Bio-Rad protein assay kit (BIO-RAD, California, CA, USA).

### 3.9. Glucose Incorporation Assay in Adipocytic Cells

In this technique mature fluorescent 3T3-F442A adipocytes grown on plates incubated for 1 h with PBS containing a solution of 1 mg/mL BSA and glucose analogue 2-NBDG at a concentration of 80 micro molars in the presence of MEA nontoxic concentrations (0.01, 0.05, 0.1 and 1 μM) were employed. Controls were also incubated and the concentrations incorporated, then after incubation the cultures were washed to remove excess 2-NBDG and fluorescent color was retained by the cells. Once this was done absorbance was measured at 485 nm, and the result of the insulin positive control was taken as 100% for the parameter set of the embodiment.

### 3.10. Statistical Analysis

All results are expressed as mean ± SD. Data were analyzed by a T-Student’s test for phenols content and antioxidant capacity and one-way ANOVA model for in vivo evaluations; followed by Dunnet’s test for multiple comparisons. Significant differences among the treatments were considered when *p* < 0.05. All analyzes were performed using GraphPad Prism 6.0 (GraphPad Software, California, CA, USA).

## 4. Conclusions

This study demonstrated that methanolic extract of *Acacciella angustissima* (MEA) showed effects on reducing the levels of glucose, insulin and improved physiological parameters, hypolipidemic effect, oxidative stress and renal damage in diabetic rats. The presence of phenolic compounds such as protocatechuic acid suggests that MEA might act in the promotion of an increased secretion of insulin by the existing β-pancreatic cells or regeneration, a mechanism that is suggested based on the literature. Thus, the experimental evidence showed in the present work, suggests that *Acaciella angustissima* might be a potential therapeutic agent for hyperglycemia and the associated diabetic disorders.

## Figures and Tables

**Table 1:** Content of phenolic compounds and antioxidant capacity of methanol and water extracts of *Acacciella angustissima*.

Sample	Extraction Solvent	Total Phenols ^1^	Condensed Tannins ^2^	Total Flavonoids ^3^	ABTS ^4^
*Acacciella angustissima*	Methanol (MEA)	121.75 ± 2.46 ^a^	0.616 ± 0.008 ^a^	4.60 ± 0.14 ^a^	70.34 ± 13.96 ^a^
	Water (W)	36.68 ± 0.70 ^b^	0.170 ± 0.04 ^b^	0.31± 0.001 ^b^	15.88 ± 1.94 ^b^

Values are presented as means ± SD (*n* = 8). ^a,b^ Mean values within a row with different superscripts were significantly different (*p* < 0.05; by the t-student test); ^1^ expressed in mg eq. gallic acid/g of sample; ^2^ expressed in mg eq. (+) catechin/g of sample; ^3^ expressed in mg eq. rutin/g sample; ^4^ Expressed in mg eq. Trolox/g of sample.

**Table 2:** Effects of oral administration of MEA on blood glucose and insulin concentration in diabetic rats.

Groups	Glucose (mg/dL)	Insulin (µU/mL)
Healthy control	86.8 ± 2.05 ^a^	28.2 ± 0.1 ^a^
25 mg/kg MEA	85.6 ± 1.27 ^a^	29.03 ± 0.3 ^a^
50 mg/kg MEA	87.47 ± 1.87 ^a^	29.45 ± 0.6 ^a^
100 mg/kg MEA	92.6 ± 2.35 ^a^	27.8 ± 0.7 ^a^
Diabetic control	372.6 ± 6.05 ^b^	7.0 ± 0.01 ^b^
Diabetic + 25 mg/kg MEA	318.9 ±3.35 ^b^	7.3 ± 0.06 ^b^
Diabetic + 50 mg/kg MEA	324.6 ±2.28 ^b^	8.7 ± 0.1 ^b^
Diabetic + 100 mg/kg MEA	146.2 ±4.03 ^a^	18.1 ± 0.02 ^a^

Values are expressed as the means ± SD of 10 rats in each group. ^a^ Significantly (*p* < 0.05) different from control diabetic group. ^b^ Significantly (*p* < 0.05) different from healthy control, where the significance was determined by one-way ANOVA followed by post hoc Dunnett’s test.

**Table 3:** Physiological and biochemical assays of healthy and diabetic rats treated with MEA

Physiological					Urine		
Groups	Body Weight (g)	Kindney Weight (g/100g B.W.)	Food Consumption (g/rat/day)	Water Consumption (ml/rat/day)	Urea (mg/dL)	Creatinine Clearance (ml/min)	Protein (mg/24 h)
Healthy Control	304 ± 20 ^a^	0.73 ± 0.1 ^a^	23 ± 5 ^a^	95 ± 19 ^a^	7.2 ± 4.0 ^a^	0.57 ± 0.01 ^a^	0.52 ± 0.03 ^a^
25 mg/Kg MEA	300 ± 12 ^a^	0.70 ± 0.3 ^a^	21.5 ± 4 ^a^	105 ± 17 ^a^	10.2 ± 7.0 ^a^	0.54 ± 0.04 ^a^	0.50 ± 0.06 ^a^
50 mg/Kg MEA	305 ± 09 ^a^	0.72 ± 0.6 ^a^	28 ± 6 ^a^	99 ± 09 ^a^	12.8 ± 4.0 ^a^	0.49 ± 0.02 ^a^	0.55 ± 0.02 ^a^
100 mg/kg MEA	297 ± 15 ^a^	0.75 ± 0.5 ^a^	22 ± 7 ^a^	117 ± 20 ^a^	13.1 ± 4.0 ^a^	0.50 ± 0.03 ^a^	0.52 ± 0.07 ^a^
Diabetic Control	272 ± 15 ^b^	1.01 ± 0.4 ^b^	46 ± 3^b^	237 ± 22^b^	50.4 ± 22.7 ^b^	0.70 ± 0.18^b^	1.05 ± 0.01 ^b^
Diabetic + 25 mg/Kg MEA	270 ± 15 ^b^	1.02 ± 0.6 ^b^	39 ± 5 ^b^	226 ± 23 ^b^	29.0 ± 0.4 ^b^	0.62 ± 0.06^b^	0.69 ± 0.05 ^ab^
Diabetic + 50 mg/Kg MEA	240 ± 17 ^b^	1.06 ± 0.5 ^b^	37 ± 4 ^b^	246 ± 18 ^b^	25.2 ± 16.4 ^b^	0.63 ± 0.09^b^	0.62 ± 0.09 ^ab^
Diabetic + 100 mg/Kg MEA	281 ± 8 ^b^	0.93 ± 0.4 ^b^	30 ± 3.1 ^a^	141 ± 11 ^a^	18.1 ± 6.5 ^a^	0.46 ± 0.09^a^	0.54 ± 0.05 ^a^

Values are expressed as the means ± SD of 10 rats in each group. ^a^ Significantly (*p* < 0.05) different from control diabetic group. ^b^ Significantly (*p* < 0.05) different from control healthy, where the significance was performed by one-way ANOVA followed by post hoc Dunnett’s test.

**Table 4:** Levels of total cholesterol, triglyceride, HDL and LDL of methanol extract of *A. angustissima* on healthy and diabetic rats.

	Determinations			
Groups	TC ^1^	TG ^2^	HDL ^3^	LDL ^4^
Healthy control	65.6 ± 4.0 ^a^	47.5 ± 2.1 ^a^	59.4 ± 6.3 ^a^	15.5 ± 1.4 ^a^
25 mg/Kg MEA	66.7 ± 1.9 ^a^	50.6 ± 2.9 ^a^	58.4 ± 5.4 ^a^	14.7 ± 2.5 ^a^
50 mg/Kg MEA	64.6 ± 2.1 ^a^	55.3 ± 3.8 ^a^	56.6 ± 2.3 ^a^	16.9 ± 2.1 ^a^
100 mg/Kg MEA	68.7 ± 3.4 ^a^	58.8 ± 3.4 ^a^	50.7 ± 3.7 ^a^	13.12 ± 1.9 ^a^
Diabetic control	104.2 ± 2.1 ^b^	72.4 ± 4.8 ^b^	47.9 ± 2.3 ^b^	51.9 ± 4.3 ^b^
Diabetic + 25 mg/Kg MEA	84.1 ± 4.4 ^b^	84.1 ± 3.6 ^b^	46.8 ± 1.9 ^b^	30.5 ± 4.3 ^b^
Diabetic + 50 mg/Kg MEA	84.9 ± 3.1 ^b^	85.1 ± 4.3 ^b^	38.3 ± 3.7 ^ab^	47.8 ± 3.9 ^b^
Diabetic + 100 mg/Kg MEA	72.9 ± 2.8 ^a^	64.5 ± 2.1 ^a^	51.2 ± 1.8 ^b^	17.6 ± 3.9 ^a^

Values are expressed as the means ± SD of 8 rats in each group. ^a^ Significantly (*p* < 0.05) different from control diabetic group. ^b^ Significantly (*p* < 0.05) different from control healthy, where the significance was performed by one-way ANOVA followed by post hoc Dunnett’s test. ^1^ TC: total cholesterol; ^2^ TG: triglyceride; ^3^ HDL: high-density lipoprotein; ^4^ LDL: low-density lipoprotein.

**Table 5:** Division of experimental animals.

	Healthy Rats				Diabetic Rats			
	Healthy Control (HC)	25 *	50 *	100 *	Diabetic Control (DC)	25 *	50 *	100 *
Base diet and water	√	√	√	√	√	√	√	√
Oral MEA		√	√	√		√	√	√
Oral Water	√				√			

*mg MEA/kg B.W./day.

## Data Availability

The data used to support the findings of this study are included within the article. The data not shown do not show statistical difference or do not contribute in a specific way in the model used.

## Abbreviations

mg	milligrams
kg	kilograms
DM	diabetes mellitus
ROS	Reactive oxygen species
MEA	methanolic extract of *Acacciella angustissima*
W	water extract of *Acacciella angustissima*
g	grams
dL	deciliters
nM	nanomolar
pH	hydrogen potential
TC	total cholesterol
TG	triglycerides
HDL	high-density lipoprotein
LDL	low-density lipoprotein
min	minutes
mL	milliliters
STZ	streptozotocin
CCr	creatinine clearance
